# Animal Welfare in Studies on Murine Tuberculosis: Assessing Progress over a 12-Year Period and the Need for Further Improvement

**DOI:** 10.1371/journal.pone.0047723

**Published:** 2012-10-26

**Authors:** Nuno Henrique Franco, Margarida Correia-Neves, I. Anna S. Olsson

**Affiliations:** 1 IBMC - Institute for Molecular and Cell Biology (Laboratory Animal Science Group), University of Porto, Porto, Portugal; 2 Life and Health Sciences Research Institute (ICVS), School of Health Sciences, University of Minho, Braga, Portugal; 3 ICVS/3B’s - PT Government Associate Laboratory, Braga/Guimarães, Portugal; Colorado State University, United States of America

## Abstract

There is growing concern over the welfare of animals used in research, in particular when these animals develop pathology. The present study aims to identify the main sources of animal distress and to assess the possible implementation of refinement measures in experimental infection research, using mouse models of tuberculosis (TB) as a case study. This choice is based on the historical relevance of mouse studies in understanding the disease and the present and long-standing impact of TB on a global scale. Literature published between 1997 and 2009 was analysed, focusing on the welfare impact on the animals used and the implementation of refinement measures to reduce this impact. In this 12-year period, we observed a rise in reports of ethical approval of experiments. The proportion of studies classified into the most severe category did however not change significantly over the studied period. Information on important research parameters, such as method for euthanasia or sex of the animals, were absent in a substantial number of papers. Overall, this study shows that progress has been made in the application of humane endpoints in TB research, but that a considerable potential for improvement remains.

## Introduction

In the study of infectious diseases, animal research continues to be of paramount importance to understand how the immune system, as well as other systems, responds to pathogens, as well as for developing and testing new drugs and vaccine candidates. By experimentally infecting standardized laboratory animals with pathogenic microorganisms, scientists generate models of infection, which can be used under controlled conditions and can be manipulated in a way that would be impossible or ethically unacceptable in the target human species.

Of note, biomedical research with animals in itself presents an ethical dilemma between the expected benefit to humans and the potential harm caused to animals. The Three Rs principle (Replacement of animal experiments with alternative approaches, Reduction of animal numbers and Refinement to improve animal welfare [1]) emerged as a way for scientists to ease this dilemma by developing research methods that decrease pain and distress. Nevertheless, the use of animals in research is still controversial, with recent voices also questioning the translational validity into humans [2], [3]. Thus, *how* research is to be conducted becomes a pertinent question from both ethical and scientific perspectives. At a point in time when research is increasingly challenged by a critical public, taking such questions seriously is essential if the scientific community is to be proactive in addressing the need for animal-based research and retaining public trust in this matter [4].

To assess how animal welfare and refinement have been considered in animal research on infection, we conducted an analysis of papers published on biomedical research on TB. This disease was chosen due to its still enormous global impact [5], [6], with implications in other diseases such as AIDS, since HIV infected individuals have increased susceptibility to develop TB. In addition, studies on TB are also expected to increase given the need a) for a more effective vaccine than Bacillus Calmette-Guérin (BCG); b) for shorter therapeutic strategies; c) for new drugs to respond to the advent of multi-drug resistant TB; d) to define surrogates of protection, and; e) for new diagnostic tests [7], [8], [9], [10]. We chose to focus on studies using mice (*Mus musculus*) as this is the most widely used species in the field [11] and is expected to remain important for TB research [12], [13].

The first potentially noxious effect of experimental TB infection in mice arises from the initial innate immune response, a phase lasting until up to three-four weeks, during which bacilli replicate exponentially (e.g. [14], [15]). In more susceptible mouse strains – like C3H, 129/Sv, A/J, CBA, DBA/2 or I/St [15], [16] – the immune response fails to control bacillary growth [17] and the disease progresses towards death, usually as a result of respiratory insufficiency [14], [18]. In more resistant mouse strains, the acquired immune response typically leads to the stabilization or slow evolution of the bacterial load [17], [19], [20], [21]. Animals have been reported to show no obvious signs of disease during this phase (e.g. [22], [23]). However, the only systematic assessment of symptomology following experimental infection with *Mycobacterium tuberculosis* reported that a sharp rise in body temperature accompanies bacillary growth during primary infection, which later on subsides [24]. Also, a transient (5 days, approximately) sickness behaviour has been reported to follow infection with BCG, accompanied by fever and weight loss, in relatively resistant mouse strains [25]. If left untreated, all *M. tuberculosis*-infected animals reach a stage of overtly symptomatic disease, with a strikingly deleterious effect on their health and wellbeing. This stage is characterized by increasingly severe clinical signs, manifested externally by respiratory distress, hunched posture, lack of grooming [26], not eating or drinking, fever and severe cachexia (e.g. [27]) and progressing to a hypokinetic irresponsive state (“moribund”) (e.g. [23]). For situations like these, where animals develop a progressively severe disease that will ultimately lead to death, implementing humane endpoints – that is, euthanizing animals to prevent unnecessary and avoidable suffering – is an important refinement [28], [29].

The aim of this study was to identify the causes of animal distress and assess the implementation of measures to improve the welfare of research animals used to investigate TB over a 12-year period.

## Results

### Trends in the Choice of Murine Models of TB

**Figure 1:**
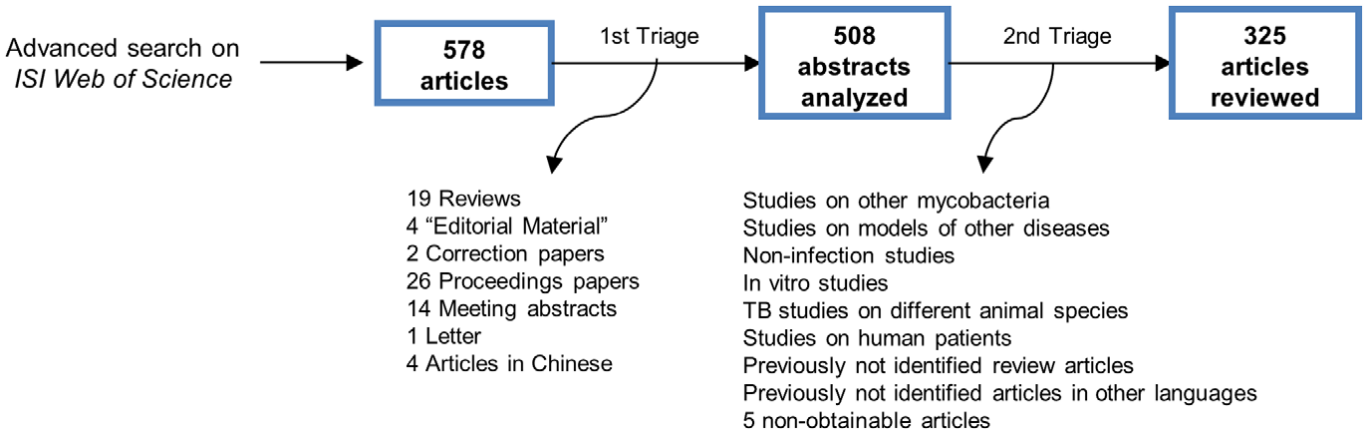
Triage of articles for analysis.

**Figure 2:**
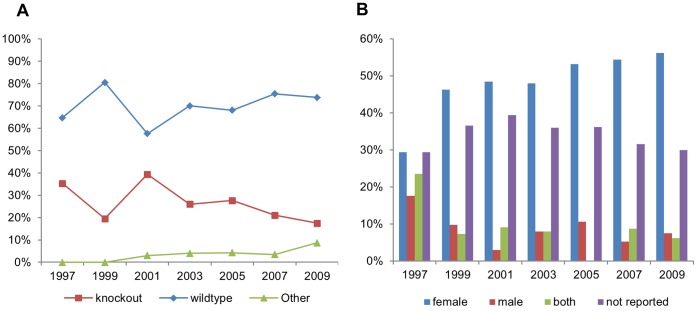
Biological characteristics of experimental animals. Reported genetic status (A) and gender (B) of mice used in experimental TB studies published between 1997 and 2009.

The number of articles per year increased almost fivefold between 1997 and 2009, being distributed by year as follows: 17 in 1997; 41 in 1999; 33 in 2001; 50 in 2003; 47 in 2005; 57 in 2007; and 80 in 2009; with a total of 325 papers for all the years (see Figure 1 for the triage process). Regarding genetic status of the animals, the majority of studies (71%, overall for all years) used non-genetically modified inbred strains. Of these (n = 231), 44% used solely C57BL/6, 23% BALB/c and 4% other inbred strains; while the remaining used two (13%) or more than two (6%) inbred strains. The remaining studies used F1 hybrids (5%) or outbred mice (6%). Of the studies using genetically modified (GM) mice in at least one of the experimental groups, 85% used knockout mice, mostly (82%) on a C57BL/6 background. The use of GM mice did not change significantly over the time period studied (Figure 2A). Two articles (out of 325) did not report the strain of mice used.

Information on the sex of animals used was not available in 34% of articles. Amongst those articles disclosing mouse sex (n = 214), the great majority (77%) reported using females (Figure 2B). No significant variation in choice of sex was found across years.

### Aerosol Exposure Overturns the Intravenous Route for Infection

**Figure 3:**
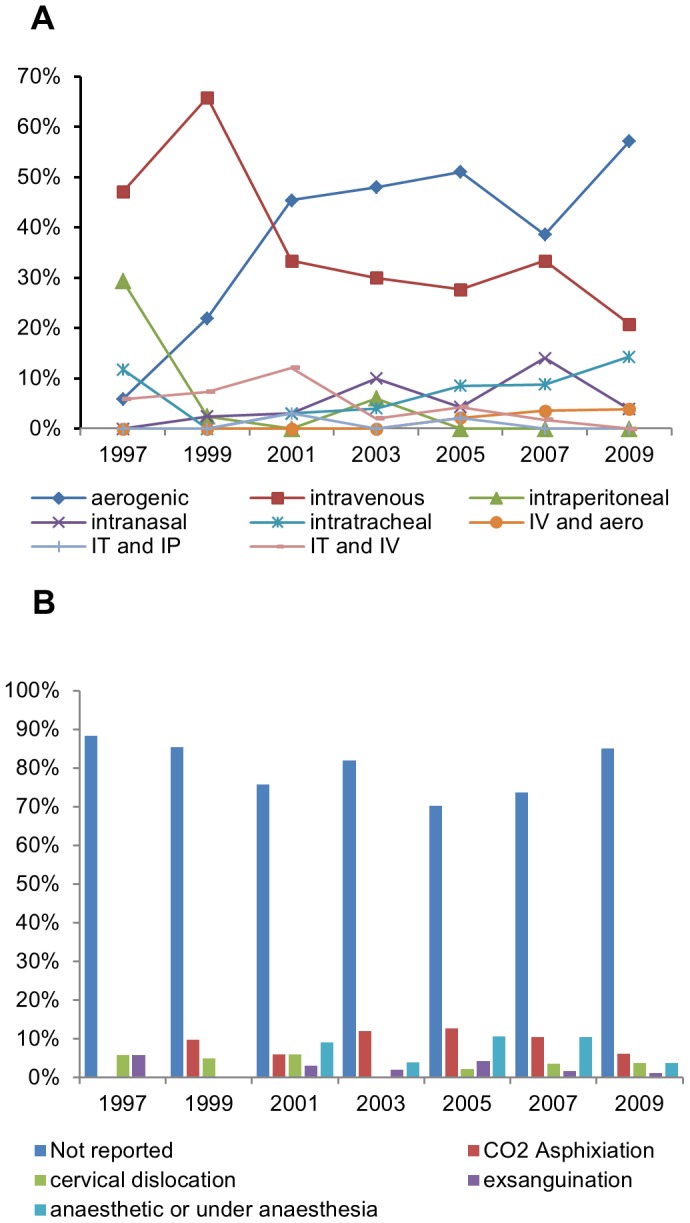
Choice of experimental methods. Route(s) of infection (A) and method of euthanasia (B) reported for TB studies using mouse models across 1997–2009.

The proportion of articles reporting to induce experimental *M. tuberculosis* infection through aerosol exposure rose significantly (linear-by-linear association p = 0.002) between 1997 and 2009 (Figure 3A). The use of the intravenous route – originally the most recurrent method – decreased (linear-by-linear association p<0.001), whereas the use of the intratracheal route remained relatively stable throughout the analysed period. The intraperitoneal route was the least chosen and not reported after 2003.

### Method for Euthanasia is Seldom Reported

The method for euthanasia was seldom reported (Figure 3B): 80% (259/325) of the articles omitted this information, with no significant differences between years. Moreover, when information on euthanasia was given (66/325), it was often incomplete and therefore difficult to interpret. For example, anaesthetic overdose was often reported without indicating the route, compound or dose, and exsanguination was frequently referred to with no indication whether under anaesthesia or not. Amidst articles reporting method of euthanasia, CO_2_ asphyxiation was the most frequently (44%; 29/66) method referred to.

### Rising Trend in Reporting Regulatory Compliance

**Figure 4:**
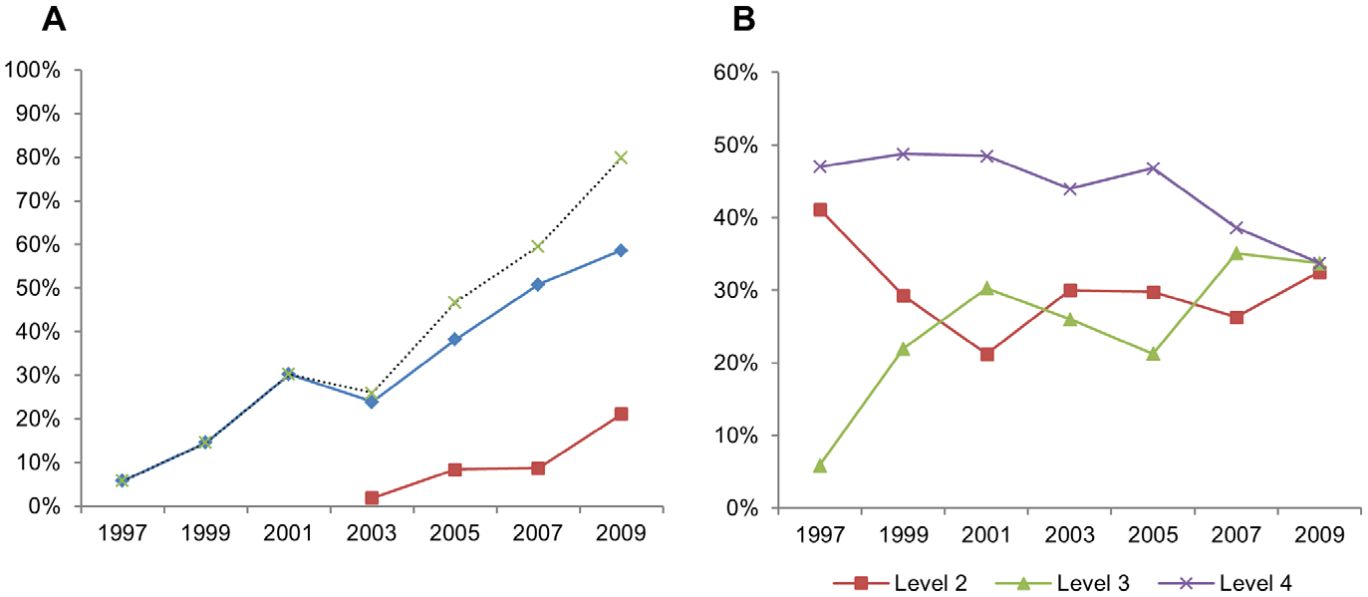
Reporting regulatory compliance and overall severity of studies across 1997–2009. In graph A, the dotted line represents the sum of articles declaring ethical approval (diamonds) and those reporting compliance with legislation or animal care guidelines (squares) for each year. Graph B illustrates the proportion of articles attributed to severity levels 2, 3 and 4 (the number of Level 1 studies – only 2 out of 325 - was negligible and is therefore not shown) for each year.

By 2009, the great majority of articles (80%) reported some type of regulatory compliance: approval by national authorities or institutional committees (59%) or compliance with national or institutional guidelines (21%). This contrasts with the panorama in 1997, when only 6% of the articles mentioned institutional approval of the experiment. This represents a significant increase in reporting regulatory compliance of any kind (linear-by-linear association p<0.001), as well as for reports of ethical approval (linear-by-linear association p<0.001) (Figure 4A).

### Most Studies Terminated before Animals become Moribund

The majority of the studies analysed were terminated before infected animals reached very severe morbidity (58%, 188/325), whereas the remaining allowed mice to reach terminal stages, with no significant variation of this proportion across years. Amidst all studies, 42% (137/325) were classified as Level 4, 28% (90/325) were classified as Level 3, 30% as Level 2 (96/325) and less than 1% (2/325) as Level 1 (Figure 4B). Among studies explicitly reporting ethical approval (123/325), 21% (26/123) were classified as Level 2, 30% (37/123) as Level 3 and 46% (59/123) as Level 4.

### Increased Implementation of Humane Endpoints in Recent Years

**Figure 5:**
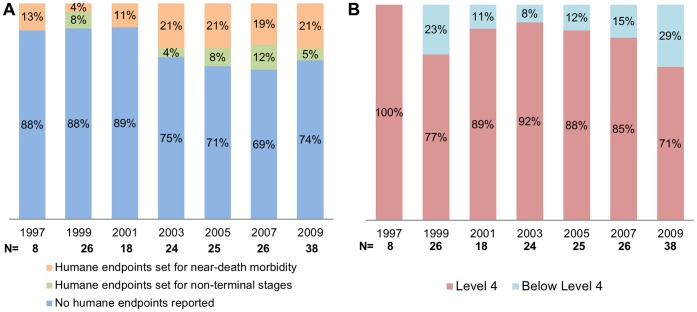
Disease stage at time-of-euthanasia in *lethal* studies. In Graph A the reported implementation of humane endpoints is shown, with these being classified as regards their effectiveness in preventing animals reaching near-death morbidity. Graph B illustrates the proportion of *lethal* studies (N = 165) classified as, or below, Level 4 severity (on account of being terminated before animals reached terminal stages), irrespective of humane endpoints being explicitly reported or not. Number of *lethal* studies distributed by year as follows: 8 in 1997; 26 in 1999; 18 in 2001; 24 in 2003; 25 in 2005; 26 in 2007; 38 in 2009.

Spontaneous death was the chosen endpoint for 66% of *lethal* studies (i.e., those conducted on very susceptible mouse strains and presenting rapidly progressive disease; or in more resistant strains with recrudescent disease; n = 165). Reported implementation of humane endpoints in *lethal* studies tended to be higher (linear by linear association p = 0.06) in later years when compared with the first years analysed, with 16% of *lethal* studies published between 1997 and 2003 (12/76) and 28% of such studies published between 2005 and 2009 (25/89) reporting the implementation of humane endpoints (Figure 5A).

The proportion of *lethal* studies explicitly reporting the implementation of humane endpoints was significantly higher (p = 0.014) for articles stating regulatory compliance (25/82) than for those that did not (12/83). However, 87% (60/70) of all *lethal* studies explicitly stating ethical approval were nevertheless classified as level 4, most of them (46/60) for allowing animals to die spontaneously, and the remaining for only implementing humane endpoints when animals showed signs of very severe morbidity (i.e., were “moribund”) (see Figure 4A). For this reason, the mere application of humane endpoints was not always sufficient for a study to be classified below the highest severity category. Of all 37 *lethal* studies reporting the use of humane endpoints, only 10 were scored as Level 3. On the other hand, if a lethal study was planned so that animals were euthanized prior to the onset of very advanced disease, humane endpoints would not be required and the study was classified as Level 3. For this reason, of all studies on *lethal* models without any explicit reference to the use of humane endpoints, 14% (18/128) were classified as Level 3. Figure 5B shows the overall proportion of *lethal* studies classified at, or below Level 4, irrespective of reporting humane endpoints.

Other types of measures to reduce animal distress were seldom reported. The most common was the administration of antibiotics in water or food instead of drug delivery through intragastric gavage, whenever this administration route was appropriate for the study. This administration route was reported in 14 out of 18 articles using antibiotics to render viable bacteria undetectable in the lung, whereas the remaining four used daily gavage. Overall, gavage for drug delivery was used in 37 studies, with variable duration, mostly in daily drug administration regimens. Also, 5 articles out of 27 describing intramuscular immunizations (most in multiple limbs), reported anaesthesia prior to this procedure. Environmental enrichment was only reported in four articles.

## Discussion

The present analysis of experimental TB research on murine models shows that the majority of published studies were terminated before animals had reached terminal stages of disease progress. Over a 12-year time period, the proportion of publications reporting ethical approval increased. However, these changes had no impact on the severity of experiments, and a considerable proportion of studies involved end-stages of infection where animals are severely affected or even allowed to die from the disease. The increasing number of studies reporting regulatory compliance nevertheless suggests a growing awareness of researchers and scientific journals to the importance of adhering to these standards. However, the incongruence of having studies reported to have been conducted according to welfare guidelines, or being ethically approved, when relevant refinement measures are apparently absent or inadequate, is in contradiction with the view of ethical appraisal of protocols as a means to ensure good practice in animal-based research [30]. The soon-to-be-enacted 2010/63/EU directive on the protection of animals used for scientific purposes emphasises the ethical evaluation process as a means to promote full consideration for the 3Rs and minimize the severity of animal experiments [31]. These results should hence serve as a note of caution for animal welfare bodies and competent authorities alike, that in order for the ethical review process to have a meaningful positive impact on animal welfare, it must be ensured that recommendations are followed-through and that proper supervision is carried out by competent personnel.

No mouse strain is capable of eradicating *M. tuberculosis* and, depending on the duration of the experiment, all are likely to develop severe disease and eventually die as a result of the disease progression [14], [15], [32] before reaching their natural average life-span [33]. The most important refinement measure for animal studies of TB is the implementation of earlier endpoints to curtail the duration and intensity of suffering. The implementation of humane endpoints was the most common refinement referred to in the analysed studies, and their application in lethal studies was more frequent in later years. However, the higher number of studies applying this refinement in the second half of the analysed period was not reflected in an overall reduction of studies categorized in the most severe level, according to the scale developed specifically for this study. As the disease resulting from experimental infection is progressively more severe, disease stage was a key consideration in the severity classification. The most severe level (Level 4, in which 42% of the studies were classified) includes studies in which animal survival is measured and animals go through end-stages and die from the disease, but also those in which researchers euthanize animals which are found ‘moribund’. This latter approach represents a scientifically relevant improvement as it allows researchers to collect tissues from animals immediately post-mortem and it avoids animals dying from secondary causes such as dehydration. Ethically this refinement measure curtails at least the very last part of the disease progress and it requires researchers to establish a protocol for animal health and welfare assessment which can potentially be adapted for earlier endpoints. But as outlined in detail elsewhere [34], euthanasia of moribund animals addresses only a small proportion of the animal welfare problems posed by this type of research since it only avoids unrelieved suffering at the moribund stage that shortly precedes death. Moreover, even in 2009 74% of the studies involving end-stages of infection did not report the use of humane endpoints.

As regards reducing the impact of experimental procedures on animal welfare, the results are more positive. The intratracheal route of infection, which we identified as the most problematic for animal welfare, was only used in 9% of all studies, while aerogenic infection was increasingly applied over the studied period, being in 2009 the most recurrently used method. This is positive from both the animal welfare and the scientific perspective, since it is not also a less invasive but also a more realistic route [11], [23], [35]. This rise may partly be due to more laboratories having access to the machine required for aerogenic infection, an apparatus that not only is expensive but also requires extra safety measures, for generating *M. tuberculosis* aerosol.

The rising trend on articles published on murine TB between 1997 and 2009 suggests a growing interest for the use of these models by the infectious diseases research community. This also indicates that non-animal models have not yet become as relevant for basic and applied research as the experimental infection of animals.

The predominance of studies using only female mice for all the years analyzed is contrary to the previously reported bias towards male animals in biomedical research [36], [37]. The most probable explanation is that researchers prefer female mice for long-lasting studies to avoid the problems with intra-group aggression in male mice, which often lead to injuries, the need to separate animals or even terminate the experiment prematurely. One third of the articles did not report which sex of mice were used, whereas almost every article reported the strain and genetic status of the mice used. These data are fairly in agreement with a survey on the quality of reporting of animals research [38], where 35% of articles analysed did not disclose mice sex, whereas all of them described the strains used. Omitting information on sex, as well as on other parameters, might cause difficulties for the replication of such studies [39]. Although sex is likely to play a less relevant role in the immune response of mice to TB when compared to other factors, it should nonetheless be considered in the interpretation of research results, since gender differences in susceptibility to TB have been reported in both humans and other animals (for mice, see [40], [41], [42]), suggesting sex to be biologically relevant in the immune response to mycobacteria [43], [44].

Information on other important methodological aspects such as details on handling and procedures, method of euthanasia as well as experimental outcome related to animal health status such as body weight variation and general animal health, were seldom reported (data not shown). Thus, one cannot exclude that some of the studies did apply refinement measures, without specifically mentioning them in the publications. In the particular case of humane endpoints, however, the wording often chosen (“animals died” or “animals were found dead”) strongly suggested that the endpoint was indeed spontaneous death rather than euthanasia. Information on refinement and on animal health may have been excluded for space constraints or for not being considered a central issue for the study, but omitting this information limits the value of the paper and the possibility to replicate and to share valid information for the design of more refined experiments [45]. Raising standards in reporting of animal experiments has been of increasing concern (e.g. [46]), and new standard guidelines [47], and a “gold standard publication checklist” for improving quality of animal studies have recently been proposed in order to improve the reproducibility of experiments, as well as facilitate systematic reviews [48]. None of these guidelines, however, stress the ethical and scientific relevance of humane endpoints, and the importance of reporting their implementation when publishing animal studies.

The proportion of articles reporting guideline compliance or ethical approval increased significantly over the 12-year period so that in 2009, 80% of articles included such a reference. These results suggest an increasing attention from authors as well as referees and editors to the ethical issue of animal research and the importance of ensuring compliance with regulatory mechanisms. Reference to ethical approval was more frequent for more severe studies (Levels 3 and 4), and the unexpected observation that the most frequently attributed severity classification for ethically approved studies was Level 4 may be an artefact of this tendency. Although it has been suggested that the main influence of ethics committee review is an increased application of refinement [49], this does not necessarily mean that one would not find highly invasive studies among those having undergone ethical approval. First, most ethics committees review both animal harm and potential human benefit [50] and would approve also severe procedures with animals if scientifically justified. Second, most animal ethics committees would probably consider the conventional application of humane endpoints a sufficient refinement of an end-stage experiment, even though we would question the efficiency of this measure in reducing animal suffering [34].

**Table 1:** Potential causes of pain and distress in studies on experimental infection with *M. tuberculosis.*

**Infection route**	
Intratracheal instillation	Surgical procedure under general anaesthesia: Inoculum delivery through an incision in the trachea, that heals in 2–3 days [52]. Two of the analysed articles reported deaths resulting from this procedure [53], [54].
Intraperitoneal injection	This injection method offers no possibility to visually confirm correct delivery, and accidental penetration of the bladder, intestine, muscular or fatty may occur [55], [56].
**Treatment administration**	
Intraperitoneal injection	Referred above.
Repeated oral gavage	Difficult procedure with risk of fluid aspiration by the lungs or perforation of oesophagic or gastric wall. Irritation, swelling and ulceration of the oesophagus from repeated dosing [56]. Unexpected deaths [57] as well as inappetence and weight loss [58] reported in experimental infection studies. Reports of increased TB susceptibility due to gavage-induced stress [59].
**Immunization**	
Footpad immunization	Immune reaction to antigen, causing swelling and inflammation *in situ*, potentially causing pain and lameness [60].
Intramuscular immunization	Painful injection that may cause mechanical trauma and potential nerve damage; immune reaction may lead to painful swelling [56], [61].
**Health Status**	
Signs of disease	Respiratory distress, hunched posture, lack of grooming [26]; failure to eat or drink, fever, severe cachexia [27]. Increasingly severe clinical signs, progressing to a hypokinetic irresponsive (“moribund”) state, culminating in death [23].

### Conclusion

Overall, this analysis demonstrates indicators of progress on ethical approval of experiments, application of humane endpoints and use of less aggressive routes of infection, but there is much to be done as regards the implementation of refinement, in particular on defining endpoints with a more relevant impact on animal wellbeing. Of course, such measures should not be taken to the expense of research quality and relevance. The best way to avoid a conflict between ethical constraints and scientific motivations is probably for scientists to be proactive and initiate a critical discussion within their own field, rather than awaiting limitations imposed from outside. In the field of experimental studies of important infections such as TB, a reassessment of the need for such a large proportion of studies to involve end-stages of the disease seems particularly pertinent.

## Materials and Methods

### Data Retrieval

We analysed articles published on murine models of *M. tuberculosis* infection published between 1997 and 2009, the first year coinciding with the date from which many journals provide digital access to full-text articles and the last with the most recent full calendar year for which journal material was available (without being subject to subscription-only embargos) at the time of data retrieval. To obtain a manageable sample size representing the full time-span, articles from every second year (i e 1997, 1999, 2001, 2003, 2005, 2007, 2009) were retrieved. This search was performed in October 2011 by applying the query *TS = ((mouse OR mice) SAME tuberculosis) AND PY = (1997 OR 1999 OR 2001 OR 2003 OR 2005 OR 2007 OR 2009)* on the *Advanced Search* option in *ISI Web of Science*® database (4.1 version, no longer accessible). Search results were refined to exclude articles written in languages other than English, proceedings papers, editorial material, meeting abstracts, reviews and correction papers. We screened the remaining articles in order to only select those reporting actual infection of mice with *M. tuberculosis*, hence excluding studies on humans or other animal models, or *in vitro* studies and infections with other mycobacteria (Figure 1).

**Table 2:** Criteria for severity categorization of experimental studies on murine tuberculosis.

Category	Criteria
**Level 1**	Induction of infection quickly followed by euthanasia, prior to any clinical signs of disease or distress (for example, studies in which animals are inoculated and euthanized shortly after for in-vitro culture of infected macrophages).
**Level 2**	Studies of infection in immunocompetent “TB resistant” animals resulting in non-lethal infection with only transient mild symptomology, and terminated before disease recrudescence; experimental groups given novel drugs or vaccines compared with positive “gold-standard” controls (e.g. BCG vaccinated animals or groups treated with currently available anti-TB drugs) resulting in sub-clinical or mild signs of the disease in all animals. Presumably lethal infections terminated before the onset of the most debilitating symptoms. The attribution of Level 2 implicates that no procedures contemplated as “Category D” or “Category E” by the CCAC are present in the study.
**Level 3**	Studies resulting in lasting deleterious effects on animal health and welfare, not alleviated by means of refinement. These include: inoculation of highly susceptible mouse strains unable to effectively control bacterial growth; use of a large inoculate size resulting in very strong immune response and/or rapidly progressive disease; infection resulting in large pneumonic areas and/or necrotizing tuberculous lesions in the lung; any experiment contemplating the following invasive procedures: daily gavaging, footpad immunization, intratracheal infection, multiple simultaneous intramuscular injections (without anaesthesia) or other procedures reported as “Category D” by the CCAC (e.g. radiation-induced sickness).
**Level 4**	All studies having spontaneous death or “moribund” state as experimental endpoints and/or resulting in severe distress non-alleviated by means of refinement.

For each article, reported information on mouse genotypes, bacterial strains used, infection route, inoculum size, protocol approval, housing conditions and method for euthanasia were retrieved, as well as complementary information on animal health status, weight variation and procedures with considerable animal welfare impact (such as daily gavage, radiation, potentially traumatic blood sampling, footpad injection, multiple intramuscular injections or intratracheal instillation) and stage of disease progression at time of euthanasia.

### Assessing Severity of Experiments

In Table 1 we briefly describe the most relevant experimental procedures with an impact on animal welfare, as well as the main welfare issues raised by the manifestation of active disease.

The severity of individual studies was classified according to a 4-level scale (Table 2) especially devised for this study, with Level 4 being the most severe. The 2010/63/EU Directive (Annex VIII) defines severity to be “determined by the degree of pain, suffering, distress or lasting harm expected to be experienced by an individual animal” [31]. For the sake of this study, severity classification was primarily based on the stage of disease animals were allowed to reach, thus taking into consideration the cumulative suffering experienced by the animal as a result of progressive disease. Additionally, the welfare impact of distressful techniques was also weighed. The Canadian Council on Animal Care Categories of Invasiveness in Animal Experiments [51] were used as a basis to build up our scale, which considered available information on the pathophysiology of the disease in several mouse strains, as well as the impact of experimental procedures on animal welfare.

The implementation of humane endpoints was assessed only for models considered to be “lethal”, i.e. all in which disease was allowed to reach severe stages, either as a result of rapidly progressive pathology in more susceptible strains or as a result of long-term evolution of disease in the other strains.

All articles were analysed at least twice by the same person (NHF), according to the defined criteria for categorization as described in Table 2.

The following considerations were taken into account for severity classification:

- The severity of each study was always the one estimated for the experimental group sustaining the most severe impact, such as non-treated groups in drug efficacy studies.
- The implementation of humane endpoints was assessed only for those models considered to be “lethal”, i.e. all in which disease was allowed to reach severe stages, either as a result of rapidly progressive pathology in more susceptible strains or as a result of long-term evolution of disease in the other strains. While studies using spontaneous death as an endpoint were categorized as a Level 4 study, the application of welfare-relevant humane endpoints [34] allowed for a Level 3 categorization.
- Unexpected deaths due to experimental procedures or other non-predicted causes were not considered to justify, *per se*, a Level 4 categorization.
- The estimated noxious impact of research procedures on welfare for the most invasive procedures – intratracheal instillation, repeated gavage or footpad immunization – was considered to justify, per se, a Level 3 categorization.
- Whenever the available information in each article was seen as insufficient for reliably assessing disease progression and outcome, such information would be complemented with historical data on disease progression and survival for the given experiment´s combination of mouse genotype, bacteria strain, inoculation route and inoculum size (unpublished data, available upon request). This information was gathered from 32 articles on the most commonly used inbred mouse strains, infected by aerosol exposure or the intravenous route. Whenever doubt persisted on classifying a study in either one or another of two possible categories, the lower degree of severity considered was attributed.
- Alongside humane endpoints in lethal studies, the implementation of other refinement measures was taken into account for the categorization of the experiments, namely drug delivery in drinking water or food (rather than through daily injections or gavage), anaesthesia, analgesia, or “gold-standard” control groups (instead of untreated groups) for drug and vaccine efficacy testing. On the other hand, the use of invasive and distressful procedures was also taken into consideration.
- Both the beneficial (“refinement”) and detrimental effect of procedures on welfare were considered. For instance, if footpad injection was performed, a non-lethal study that would otherwise be classified as Level 2 would be categorized as Level 3. On the other hand, if animals were anaesthetized before undergoing intramuscular injection on multiple limbs, the latter would not be accounted as distress-inducing procedure.

### Statistical Analysis

Chi-square tests were applied to determine association between variables. The Mantel-Haenszel linear-by-linear association test was to assess linear relationship between ordinal variables. The level of significance used for all tests was 0.05. The statistical analysis was performed using the software SPSS®.

## Supporting Information

PRISMA 2009 Checklist S1 Checklist of the information regarding the systematic review data and their position in the manuscript.
(DOC)

PRISMA 2009 Flow Diagram S1 Flow diagram representing the article retrieval and triage process.
(DOC)
