# Supplementary material for: Animal Welfare in Studies on Murine Tuberculosis: Assessing Progress over a 12-Year Period and the Need for Further Improvement
Source: PLoS One. 2012 Oct 26;7(10):e47723. doi: 10.1371/journal.pone.0047723 (PMC3482232; doi:10.1371/journal.pone.0047723)
Supplement: PRISMA 2009 Flow Diagram S1 — Flow diagram representing the article retrieval and triage process. (DOC) [file pone.0047723.s002.doc]

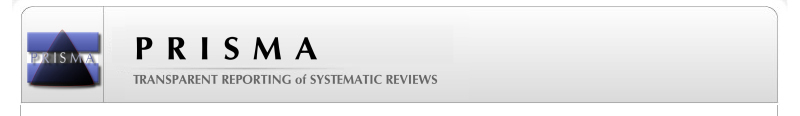
**PRISMA 2009 Flow Diagram**

**Screening**

**Included**

**Eligibility**

**Identification**

Records identified through database searching
(n = 578 )

Additional records identified through other sources
(n = 0 )

Records after duplicates removed
(n = 578)

Records screened
(n =578 )

Records excluded
(n = 70)

Full-text articles assessed for eligibility
(n = 508 )

Full-text articles excluded, with reasons
(n = 183 )

Studies included in qualitative synthesis
(n = 325 )

Studies included in quantitative synthesis (meta-analysis)
(n = 325 )
